# Socio-demographic factors associated with hunger among food pantry users in Eastern Massachusetts

**DOI:** 10.1017/jns.2022.118

**Published:** 2023-04-27

**Authors:** Alyson Codner, Rachel M. Zack, Xinyang Liu, Candice Bangham, Eva Nelson, Jacqueline Milton Hicks, Jacey A. Greece

**Affiliations:** 1 Department of Community Health Sciences, Boston University School of Public Health, 801 Massachusetts Avenue, 4th Floor, Boston, MA 02118, USA; 2 Greater Boston Food Bank, 70 S Bay Ave, Boston, MA 02118, USA; 3 Department of Biostatistics, Boston University School of Public Health, 801 Massachusetts Avenue, 3rd Floor, Boston, MA 02118, USA

**Keywords:** Charitable food assistance, Economic hardship, Food insecurity, Food pantry, Hunger

## Abstract

To assess the determinants of hunger among food pantry users, the present study used a cross-sectional survey that included a modified Household Hunger Scale to quantify hunger. Mixed-effects logistic regression models were used to assess the relationship between hunger categories and various household socio-demographic and economic characteristics, such as age, race, household size, marital status and experience of any economic hardship. The survey was administered to food pantry users from June 2018 to August 2018 at various food pantries across Eastern Massachusetts with 611 food pantry users completing the questionnaire at any of the 10 food pantry sites. One-fifth (20⋅13 %) of food pantry users experienced moderate hunger and 19⋅14 % experienced severe hunger. Food pantry users who were single, divorced or separated; had less than a high school education; working part-time, unemployed or retired; or, who earned incomes less than $1000 per month were most likely to experience severe or moderate hunger. Pantry users who experienced any economic hardship had 4⋅78 the adjusted odds of severe hunger (95 % CI 2⋅49, 9⋅19), which was much larger than the odds of moderate hunger (AOR 1⋅95; 95 % CI 1⋅10, 3⋅48). Younger age and participation in WIC (AOR 0⋅20; 95 % CI 0⋅05–0⋅78) and SNAP (AOR 0⋅53; 95 % CI 0⋅32–0⋅88) were protective against severe hunger. The present study illustrates factors affecting hunger in food pantry users, which can help inform public health programmes and policies for people in need of additional resources. This is essential particularly in times of increasing economic hardships recently exacerbated by the COVID-19 pandemic.

## Introduction

Food insecurity is a measure widely used by community-based organisations as well as federal and local agencies for surveillance, programme development and programme evaluation because it is more straightforward to measure as an economic condition rather than hunger, an individual physiological condition^(1,2)^. Food insecurity is defined as an ‘economic and social condition of limited or uncertain access to adequate food’ and is associated with a number of adverse health effects including inadequate nutrient intake, chronic conditions and poor general and mental health in adults and children^(1–4)^. In 2020, 13⋅8 million (10⋅5 %) of households in the US experienced food insecurity for at least some part of the year^(5)^. Food insecurity continues to be an area of public health focus especially given the shifting context resulting from the COVID-19 pandemic^(6)^. Changes in life events, such as loss of a job or unexpected expenses, often precipitate a change in food security status and that combined with the economic shutdowns during the pandemic impacted food insecurity in the US^(7)^.

Food insecurity is categorised and defined as very low food security (often not enough to eat), low food security (sometimes not enough to eat), marginal food security (enough food to eat, but not always the kinds of food the household wanted to eat) and high food security (enough of the kinds of food the household wanted to eat)^(1)^. While the concepts are related, food insecurity is an economic condition of inadequate access to healthy food, while hunger is the physiological condition that can result from experiencing food insecurity^(1)^. Hunger is a more severe form of food insecurity that is heavily influenced by several social and economic determinants of health and is rarely researched as an outcome of interest given the complexities in defining and quantifying it^(1,2)^.

Accordingly, research has focused on understanding risk factors for food insecurity to inform approaches to address hunger. Some of the well-documented socio-demographic and economic determinants of food insecurity include income, employment status, disability, household size and makeup, race, and marital status^(5,8,9)^. For instance, Black/African American and Hispanic people experience higher rates of food insecurity than their white counterparts; individuals who are single experience more food insecurity than married couples; and households with children report more food insecurity than households without children^(5,8)^.

The onset and prolonged impacts of the COVID-19 pandemic have exacerbated food insecurity especially for those populations already at risk^(5)^. Households that were previously food insecure have been heavily impacted by the pandemic due to disproportionate loss of income, quarantining, social distancing, school closures and unequal disease burden^(7,10)^. Poverty rates increased due to COVID-19 and food insecurity largely remained constant (10⋅5 % of households experienced food insecurity and 3⋅9 % experienced very low food security) yet did increase in some populations including households with children or with a Black or Hispanic head of household^(5,11)^. This is consistent with findings pre-pandemic that lower income levels and unemployment strongly contribute to food insecurity^(12–14)^.

Many charitable and governmental food assistance programmes expanded to address the increased demand during the pandemic^(15)^. Federal food assistance programmes such as the Supplemental Nutrition Assistance Program (SNAP) and Women, Infants and Children program (WIC) aim to address hunger, with eligibility determined by income, work status, immigration status and the presence of children in the household^(16,17)^. The average monthly number of SNAP users increased by 11⋅7 % from 2019 to 2020, while charitable food assistance programmes, like food pantries, saw a 50 % increase in overall usage from December 2019 to December 2020^(18,19)^.

While these food assistance programmes are helpful, the benefits are often inadequate to lift people out of food insecurity and hunger. Access to and eligibility for such programmes may be disproportionately distributed across populations resulting in persistent gaps in food security^(17,20)^. Additionally, households may face several barriers to utilising these resources such as the complex application process, stigma, lack of knowledge about the application process and failure to meet specific eligibility criteria such as income limits, work requirements and citizenship status^(21,22)^. Even though charitable food assistance resources, such as food banks and food pantries, attempt to fill these gaps, they are often only used by those facing more severe food insecurity and are intended to provide immediate, short-term, emergency food relief^(23,24)^. Increasing adequate programme availability for those with the greatest need necessitates understanding the determinants of food insecurity and hunger, and measuring those outcomes, to establish more comprehensive, long-term approaches.

Hunger is an individual-level physiological condition that may result in food insecurity while food insecurity is a broader term with various root causes^(1)^. Therefore, providing food alone does not address the root causes of food insecurity. For example, it does not help someone secure a job that provides a living wage or resolve domestic abuse situations, homelessness or medical expenses. Providing food, however, results in relieving of an acute hunger problem and stabilising the individual so they can be better equipped to address the root causes of their food insecurity. Food assistance programmes that provide food, such as food banks and food pantries, are helping to relieve hunger and accordingly is the outcome of focus.

The present study explores determinants of hunger among food pantry users in Eastern Massachusetts to better understand the populations who are already using food pantries but remain in need of additional food assistance. In the first four months of the pandemic, food insecurity in the US increased by 26 % with Massachusetts experiencing the greatest increase (47 %)^(25,26)^. There are clear determinants of hunger in people who already use food assistance programmes, some of which have been exacerbated by the COVID-19 pandemic. Although the present study was conducted prior to the COVID-19 pandemic, its findings can lend insight to the populations that would be most positively impacted by ongoing public health efforts to address hunger during the pandemic including understanding the impact of determinants on moderate and severe hunger and having a brief scale to measure hunger.

## Methods

### Study sample and data collection

**Fig. 1.**
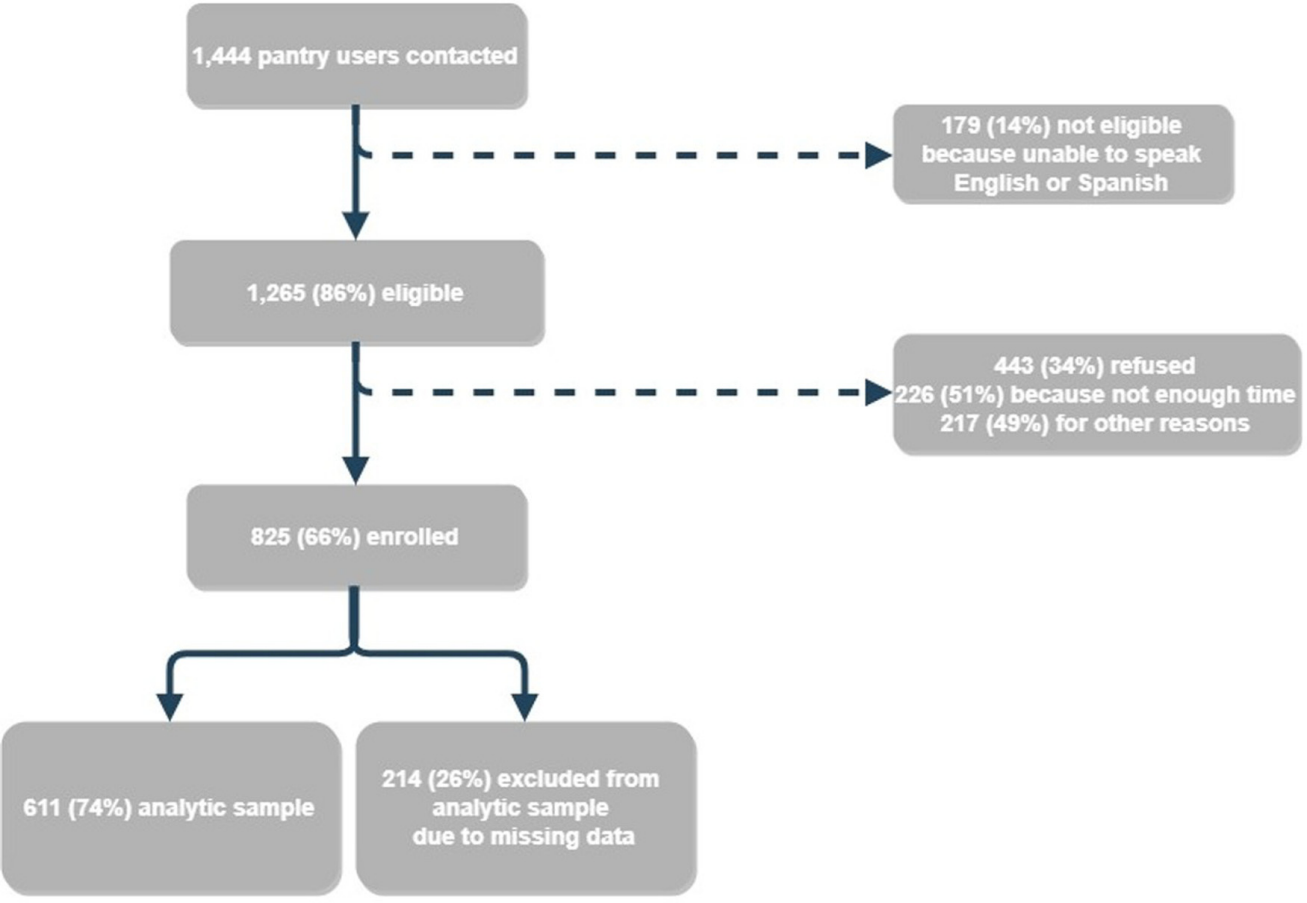
Hunger study enrolment flowchart, food pantry users in ten food pantries in Eastern Massachusetts, June 2018–August 2018.

The present study was conducted from June 2018 through August 2018 in partnership with The Greater Boston Food Bank (GBFB) and 10 of their partner food pantries in Eastern Massachusetts, where food pantry users were recruited as study participants. Study sites were chosen based on volume (serving at least 1000 households per month in 2017) and differed greatly in the number of study participants (range 10 to 276) and demographics. Food pantry users received a baseline survey if they visited one of the study sites and met the following criteria: (1) were at least 18 years old or older; (2) were physically and mentally capable of completing the survey; (3) spoke English or Spanish; and (4) were not planning on moving within the next 3 months. Of the 1444 people that were approached to be in the study, 825 (57⋅1 %) agreed to participate (Fig. 1). Reasons for refusal included not having enough time, being in a rush, not speaking English or Spanish and/or not understanding the study. Participants were given a $10 gift card as compensation for their time. This study was conducted according to the guidelines laid down in the Declaration of Helsinki and all procedures involving human subjects were approved by the Boston University Institutional Review Board (study #H-37567). Verbal informed consent was obtained from all subjects and verbal consent was witnessed and formally recorded.

The survey took approximately 15 min to complete. After providing consent, participants completed the survey at the food pantry on iPad tablets through the method of their choice, either interviewer-administration (66 %) or self-administration (34 %), with 648 individuals completing the survey in English and 172 in Spanish. The survey included questions on demographics, household characteristics, use of food assistance programmes, household economic hardship and hunger.

### Measures

The outcome, hunger, was assessed using a modified version of the validated Household Hunger Scale (HHS), which has been used in cross-cultural settings for the monitoring and evaluation of hunger^(27–30)^. The HHS is a shortened version of the Household Food Insecurity Access Scale, which has been used in high need populations in the US^(31)^. The HHS was used in this study given it was conducted in a high need group representing a wide variety of cultural backgrounds and known to have a very high prevalence of food insecurity. The tool was administered in a time-constrained, fast-paced environment at food pantries and accordingly a shorter tool to quickly measure severe food need was necessary.

The modified scale consisted of the following questions: (1) ‘In the past 30 days, how often was there ever no food to eat of any kind in your house because of lack of resources to get food?’; (2) ‘In the past 30 days, how often did you or any household member go to sleep at night hungry because there was no enough food?’; and (3) ‘In the past 30 days, how often did you or any household member go a whole day and night without eating anything at all because there was no enough food?’. Response options included ‘never (0 times)’, ‘rarely (1–2 times)’, ‘sometimes (3–10 times)’, and ‘often (10+ times)’. Response options of ‘sometimes’ and ‘often’ were given a score of 2; ‘rarely’ was given a score of 1 and ‘never’ a score of 0 for each question^(27–30)^. Per HHS protocol, the scores for each question were summed (range score 0–6) to create a hunger indicator score^(27–30)^. This score was further categorised into an ordinal outcome as little to no hunger in the household (score = 0–1), moderate hunger in the household (score = 2–3) and severe hunger in the household (score = 4–6)^(27–30)^. A binary hunger variable was also created (defined as a hunger indicator score of 2 or greater) to examine the presence of any hunger, regardless of severity. Binary results mirror the categorical results and therefore are not presented in tables.

Socio-demographic variables examined were age, race, gender, after-tax household monthly income, educational attainment, marital status, household composition, use of food assistance and recent experience of economic hardship. Gender was self-reported with the following pre-defined response options: male, female, other and prefer not to answer. Due to small sample sizes, other and prefer not to answer are not shown in this analysis. Individuals self-reported their race according to the pre-defined response options of Hispanic, non-Hispanic White, non-Hispanic Black and non-Hispanic Other. Household composition and size was calculated from questions on the number of children (<18 years), adults (≥18–<65 years) and seniors (≥65 years) in the household. Household size was recategorised from a continuous number into categorical variable. Food assistance use included data on SNAP, WIC and food pantry use in the past 30 d. Economic hardship was evaluated by asking participants to select any economic hardships they or anyone in their household experienced in the past 3 months. Participants could select all that apply from the following options: (1) significant out-of-pocket medical expenses, (2) lost a job, (3) had work hours and/or pay reduced, (4) divorce, (5) received a foreclosure or eviction notice, (6) death of primary breadwinner, (7) death of other family member, (8) a family-owned business had financial difficulty, (9) major home repairs, (10) interest/late fees from payday loans, (11) loan repayment from debt collectors, (12) legal expenses and (13) other. The binary economic hardship variable was created as experiencing at least one of the economic hardships.

### Data analysis

Frequencies and percentages are presented for categorical variables and means and standard deviations for continuous variables. Pearson's *χ*^2^ were calculated and *P*-values were considered statistically significant at an alpha level of 0⋅05. Mixed-effects models were used because demographics of pantry users differed by food pantry site, specifically by educational attainment, race and age. These models adjusted for food pantry site as a random effect while all other covariates were controlled for as fixed effects. Due to the fact that there was missing data in the full dataset, the distribution of variables was examined across all missing data patterns to diagnose the missing data mechanism. No relationship was found between the missingness of the data and the values, indicating that the data were missing completely at random (MCAR). The analytic sample represents participants who had responses to all questions under investigation. All analyses were performed using SAS® software version 9⋅4 (SAS Institute Inc., Cary, NC, USA).

## Results

### Participant characteristics

Of the 611 participants, the majority were female (72⋅50 %), aged 50 years or older (60⋅89 %), did not have children in their household (57⋅94 %), were non-Hispanic Black (26⋅51 %) or Hispanic (27⋅82 %), had a high school or some college education (60⋅23 %), were not married (72⋅50 %), lived in a household with two or more people (70⋅7 %), did not work full-time (84⋅45 %) and earned less than $1500 per month (72⋅18 %). More than half of participants had experienced an economic hardship in the past 3 months (60⋅39 %), were enrolled in SNAP (54⋅83 %) and had used a pantry in the past 30 d (87⋅73 %). Roughly two-thirds (60⋅72 %) experienced little to no hunger, 20⋅13 % experienced moderated hunger and 19⋅15 % experienced severe hunger (Table 1).

**Table 1.** Hunger study participant characteristics by hunger level, food pantry users in ten food pantries in Eastern Massachusetts, June 2018–August 2018, *n* 611

Variable	Overall *N* 611	Little to no hunger^a^ *N* 371	Moderate hunger^a^ *N* 123	Severe hunger^a^ *N* 117	*P*
	*N* (%)	*N* (%)	*N* (%)	*N* (%)	
Age^b^ [mean (std)]	54⋅00 (15⋅5)				
18–<30	48 (7⋅86)	19 (5⋅12)	13 (10⋅57)	16 (13⋅68)	**<0**⋅**01**
30–<40	87 (14⋅24)	53 (14⋅29)	18 (14⋅63)	16 (13⋅68)	
40–<50	104 (17⋅02)	58 (15⋅63)	22 (17⋅89)	24 (20⋅51)	
50–<60	177 (28⋅97)	100 (26⋅95)	38 (30⋅89)	39 (33⋅33)	
60–<65	70 (11⋅46)	47 (12⋅67)	14 (11⋅38)	9 (7⋅69)	
65 and older	125 (20⋅46)	94 (25⋅34)	18 (14⋅63)	13 (11⋅11)	
Gender					0⋅07
Male	168 (27⋅50)	104 (28⋅03)	25 (20⋅33)	39 (33⋅33)	
Female	443 (72⋅50)	267 (71⋅97)	98 (79⋅67)	78 (66⋅67)	
Race/Ethnicity^c^					0⋅08
Non-Hispanic White	230 (37⋅64)	156 (42⋅05)	36 (29⋅27)	38 (32⋅48)	
Non-Hispanic Black	162 (26⋅51)	91 (24⋅53)	38 (30⋅89)	33 (28⋅21)	
Non-Hispanic Other	49 (8⋅02)	23 (6⋅20)	12 (9⋅76)	14 (11⋅97)	
Hispanic	170 (27⋅82)	101 (27⋅22)	37 (30⋅08)	32 (27⋅35)	
Educational attainment					**0**⋅**05**
College Graduate (4 years)	93 (15⋅22)	69 (18⋅60)	12 (9⋅76)	12 (10⋅26)	
High school or some college	368 (60⋅23)	218 (58⋅76)	74 (60⋅16)	76 (64⋅96)	
Less than high school	150 (24⋅55)	84 (22⋅64)	37 (30⋅08)	29 (24⋅79)	
Marital status					**<0**⋅**01**
Married/Living with partner	168 (27⋅50)	119 (32⋅08)	26 (21⋅41)	23 (19⋅66)	
Separated or Divorced/Widowed	225 (36⋅82)	142 (38⋅27)	45 (36⋅59)	38 (32⋅48)	
Single/Never married	218 (35⋅68)	110 (29⋅65)	52 (42⋅28)	56 (47⋅86)	
Work status					**0**⋅**03**
Full-time (>35 h)	95 (15⋅55)	64 (17⋅25)	12 (9⋅76)	19 (16⋅24)	
Part-time (<35 h)	118 (19⋅31)	63 (16⋅98)	30 (24⋅39)	25 (21⋅37)	
Homemaker	50 (8⋅18)	33 (8⋅89)	11 (8⋅94)	6 (5⋅13)	
Unemployed	95 (15⋅55)	52 (14⋅02)	21 (17⋅07)	22 (18⋅80)	
Disabled	150 (24⋅55)	84 (22⋅64)	31 (25⋅20)	35 (29⋅91)	
Retired	94 (15⋅38)	71 (19⋅14)	15 (12⋅20)	8 (6⋅84)	
Other	9 (1⋅47)	4 (1⋅08)	3 (2⋅44)	2 (1⋅71)	
Monthly household income^d^					**0**⋅**01**
$2000 or more	81 (13⋅26)	62 (16⋅71)	11 (8⋅94)	8 (6⋅84)	
$1500 to $1999	89 (14⋅57)	58 (15⋅63)	16 (13⋅01)	15 (12⋅82)	
$1000 to $1499	149 (24⋅39)	96 (25⋅88)	24 (19⋅51)	29 (24⋅79)	
$500 to $999	181 (29⋅62)	93 (25⋅07)	48 (39⋅02)	40 (34⋅19)	
Less than $500	111 (18⋅17)	62 (16⋅71)	24 (19⋅51)	25 (21⋅37)	
Household size^e^					0⋅08
1	179 (29⋅30)	118 (31⋅81)	26 (21⋅14)	35 (29⋅91)	
2–3	234 (38⋅30)	147 (39⋅62)	46 (37⋅40)	41 (35⋅04)	
4–5	143 (23⋅40)	79 (21⋅29)	38 (30⋅89)	26 (22⋅22)	
>5	55 (9⋅00)	27 (7⋅28)	13 (10⋅57)	15 (12⋅82)	
Household composition^f^					
Children in household	257 (42⋅06)	144 (38⋅81)	64 (52⋅03)	49 (41⋅88)	**0**⋅**04**
Seniors in household	182 (29⋅79)	127 (34⋅23)	32 (26⋅02)	23 (19⋅66)	**<0**⋅**01**
Food assistance use in the past 30 d					
SNAP	335 (54⋅83)	202 (54⋅45)	76 (61⋅79)	57 (48⋅72)	0⋅12
WIC	57 (9⋅33)	39 (10⋅51)	14 (11⋅38)	4 (3⋅42)	**0**⋅**05**
Food pantry^g^	536 (87⋅73)	338 (91⋅11)	107 (86⋅99)	91 (77⋅78)	**<0**⋅**01**
Any economic hardship^h^	369 (60⋅39)	192 (51⋅57)	81 (65⋅85)	96 (82⋅05)	**<0**⋅**01**

Analyses were conducted using frequencies and Pearson's *χ*^2^ statistical test significance 0⋅05 indicated by boldface.

a Hunger categories were defined as little to no hunger in the household (HHS score 0–1), moderate hunger in the household (HHS score 2–3) and severe hunger in the household (HHS 4–6) according to the HHS score.

b Age categories were created based on pre-established age definitions from the US Census.

c Individuals self-reported their race according to the pre-defined response options of Hispanic, non-Hispanic White, non-Hispanic Black and non-Hispanic Other.

d Income categories were created based on open-ended responses for annual/monthly income.

e Household size categories were created based on the open-ended responses of the number of people in household.

f Household composition for both children and seniors in the household were defined as at least one or more in the household.

g Food pantry use was defined as use of a food pantry in the past 30 d aside from the visit of survey completion.

h Economic hardship was defined as experiencing at least one of the following in the past 3 months: medical expenses, job loss, reduced pay/hours, divorce, rent or utilises, foreclosure/eviction notice, death of a family member or breadwinner, business financial difficulty, home repairs, loan repayment or loan late fees, legal expenses or other hardship.

### Unadjusted mixed-effects models

Unadjusted mixed-effects models examined determinants of hunger controlling for food pantry sites (Table 2). Compared with food pantry users who worked full-time, those who were disabled (odds ratio (OR) 1⋅96, 95 % CI 1⋅07, 3⋅60) or retired (OR 3⋅59, 95 % CI 1⋅43, 9⋅02) were more likely to experience moderate hunger. The magnitude of these associations was larger among these two groups for severe hunger (OR 3⋅23, 95 % CI 1⋅83–5⋅69; OR 4⋅20, 95 % CI 3⋅02, 5⋅86, respectively). Individuals who were homemakers (OR 3⋅48, 95 % CI 1⋅82, 6⋅64) or working part-time (OR 3⋅32, 95 % CI 1⋅19, 8⋅82) were significantly associated with severe hunger only. Incomes less than $500 a month had 2⋅48 times the odds of moderate hunger (95 % CI 1⋅24, 4⋅97) and 3⋅12 times the odds of severe hunger (95 % CI 1⋅48, 6⋅60) when compared with those with an income of $2000 or more a month. Incomes ranging from $500 to $999 a month (OR 2⋅22, 95 % CI 1⋅56, 3⋅12) and $1500 to $1999 a month (OR 2⋅85, 95 % CI 1⋅12, 7⋅22) were significantly associated with severe hunger. Marital status of separated or divorced/widowed were associated with both moderate (OR 2⋅00, 95 % CI 1⋅26, 3⋅18) and severe (OR 2⋅59, 95 % CI 1⋅90, 3⋅52) hunger when compared to individuals who were married or living with a partner. Age, WIC use in the past 30 d and food pantry use in the past 30 d were all protective against severe hunger, but only age was significantly protective against moderate hunger (OR 0⋅98, 95 % CI 0⋅97, 0⋅99). The odds of severe hunger among food pantry users who experienced any economic hardship in the past 30 d was 4⋅26 (95 % CI 2⋅32, 7⋅82) compared with those who did not experience any economic hardship, which was even higher than the odds of experiencing moderate hunger (OR 1⋅79, 95 % CI 1⋅03, 3⋅10) (Table 2).

**Table 2.** Unadjusted mixed-effects models assessing associations between characteristics and ordinal hunger in food pantry users in ten pantries in Eastern Massachusetts, *n* 611

Variable	Moderate hunger vs. little to no hunger^a^^,^^b^		Severe hunger vs. little to no hunger^a^^,^^b^	
	Odds ratio (95 % CI)	*P*	Odds ratio (95 % CI)	*P*
Age^c^	0⋅98 (0⋅97, 0⋅99)	**<0⋅01**	0⋅97 (0⋅96, 0⋅98)	**<0**⋅**01**
Gender				
Male	Ref^d^		Ref^d^	
Female	1⋅44 (0⋅91, 2⋅28)	0⋅12	0⋅78 (0⋅61, 1⋅00)	0⋅05
Race/Ethnicity^e^				
Non-Hispanic White	Ref^d^		Ref^d^	
Non-Hispanic Black	1⋅56 (0⋅61, 3⋅98)	0⋅35	1⋅79 (0⋅51, 6⋅29)	0⋅37
Non-Hispanic Other	1⋅11 (0⋅60, 2⋅06)	0⋅75	1⋅19 (0⋅69, 2⋅06)	0⋅54
Hispanic	0⋅83 (0⋅44, 1⋅55)	0⋅56	0⋅57 (0⋅40, 0⋅80)	**<0**⋅**01**
Educational attainment				
College Graduate (4 years) or more	Ref^d^		Ref^d^	
High school or some college	1⋅68 (0⋅86, 3⋅31)	0⋅13	1⋅90 (1⋅10, 3⋅27)	**0**⋅**02**
Less than high school	2⋅02 (1⋅07, 3⋅81)	**0**⋅**03**	1⋅79 (1⋅15, 2⋅78)	**<0**⋅**01**
Marital status				
Married/Living with partner	Ref^d^		Ref^d^	
Separated or Divorced/Widowed	2⋅00 (1⋅26, 3⋅18)	**<0**⋅**01**	2⋅59 (1⋅90, 3⋅52)	**<0**⋅**01**
Single/Never married	1⋅39 (0⋅98, 1⋅96)	0⋅07	1⋅39 (0⋅87, 2⋅22)	0⋅16
Work status				
Full-time (>35 h)	Ref^d^		Ref^d^	
Part-time (<35 h)	1⋅55 (0⋅78, 3⋅08)	0⋅21	3⋅23 (1⋅19, 8⋅82)	**0**⋅**02**
Homemaker	1⋅53 (0⋅91, 2⋅55)	0⋅12	3⋅48 (1⋅82, 6⋅64)	**<0**⋅**01**
Unemployed	1⋅41 (0⋅56, 3⋅59)	0⋅47	1⋅51 (0⋅58, 3⋅91)	0⋅40
Disabled	1⋅96 (1⋅07, 3⋅60)	**0**⋅**03**	3⋅23 (1⋅83, 5⋅69)	**<0**⋅**01**
Retired	3⋅59 (1⋅43, 9⋅02)	**<0**⋅**01**	4⋅20 (3⋅02, 5⋅86)	**<0**⋅**01**
Other	0⋅79 (0⋅36, 1⋅74)	0⋅06	2⋅37 (0⋅76, 7⋅38)	0⋅14
Monthly household income^e^				
$2000 or more	Ref^d^		Ref^d^	
$1500 to $1999	1⋅76 (0⋅78, 3⋅99)	0⋅17	2⋅85 (1⋅12, 7⋅22)	**0**⋅**03**
$1000 to $1499	1⋅43 (0⋅68, 3⋅00)	0⋅34	1⋅95 (0⋅83, 4⋅59)	0⋅13
$500 to $999	1⋅34 (0⋅54, 3⋅33)	0⋅53	2⋅22 (1⋅56, 3⋅12)	**<0**⋅**01**
Less than $500	2⋅48 (1⋅24, 4⋅97)	**0**⋅**01**	3⋅12 (1⋅48, 6⋅60)	**<0**⋅**01**
Household size^f^				
1	Ref^d^		Ref^d^	
2–3	1⋅45 (0⋅95, 2⋅22)	0⋅09	1⋅17 (0⋅79, 1⋅74)	0⋅45
4–5	1⋅34 (0⋅67, 2⋅71)	0⋅41	1⋅87 (0⋅99, 3⋅54)	0⋅05
>5	0⋅73 (0⋅52, 1⋅04)	0⋅08	1⋅09 (0⋅73, 1⋅63)	0⋅68
Household composition^g^				
Children in the household	1⋅58 (1⋅26, 1⋅98)	**<0**⋅**01**	1⋅08 (0⋅79, 1⋅48)	0⋅62
Seniors in the household	0⋅74 (0⋅45, 1⋅23)	0⋅25	0⋅49 (0⋅30, 0⋅80)	**<0**⋅**01**
Food assistance use in the past 30 d^h^				
SNAP	1⋅25 (0⋅96, 1⋅62)	0⋅10	0⋅77 (0⋅50, 1⋅17)	0⋅22
WIC	1⋅09 (0⋅72, 1⋅66)	0⋅68	0⋅36 (0⋅14, 0⋅72)	**<0**⋅**01**
Food pantry^i^	0⋅77 (0⋅49, 1⋅21)	0⋅26	0⋅36 (0⋅22, 0⋅58)	**<0**⋅**01**
Any economic hardship^j^	1⋅79 (1⋅03, 3⋅10)	**0**⋅**04**	4⋅26 (2⋅32, 7⋅82)	**<0**⋅**01**

Analyses were conducted using mixed-effects models for an ordinal outcome, with significance 0⋅05 indicated by boldface. The reference group for these analyses is little to no hunger in the household.

a Hunger categories were defined as little to no hunger in the household (HHS score 0–1), moderate hunger in the household (HHS score 2–3) and severe hunger in the household (HHS 4–6) according to the HHS score.

b Food pantry site was controlled for as a random effect in these models.

c Age categories were created based on pre-established age definitions from the US Census.

d Socio-demographic categories that previous studies have found to be protective against food insecurity were used as the reference category.

e Individuals self-reported their race according to the pre-defined response options of Hispanic, non-Hispanic White, non-Hispanic Black and non-Hispanic Other. Income categories were created based on open-ended responses for annual/monthly income.

f Household size categories were created based on the open-ended responses of the number of people in household.

g Household composition for both children and seniors in the household were defined as at least one or more in the household.

h Food assistance use (SNAP, WIC, food pantry) were defined as any use in the past 30 d, with no use as the comparison.

i Food pantry use defined as use of a food pantry in the past 30 d aside from the visit of survey completion.

j Economic hardship defined as experiencing at least one of the following in the past 3 months: medical expenses, job loss, reduced pay/hours, divorce, rent or utilises, foreclosure/eviction notice, death of a family member or breadwinner, business financial difficulty, home repairs, loan repayment or loan late fees, legal expenses or other hardship.

### Adjusted mixed-effects models

Mixed-effects models adjusting for covariates are shown in Table 3. Pantry users with less than a high school level of education had 2⋅37 times the odds of moderate hunger (95 % CI 1⋅11, 5⋅06) compared with college graduates. This association was attenuated for severe hunger, with OR 1⋅97 (95 % CI 1⋅12, 3⋅47). Marital status was significantly associated with both moderate and severe hunger. The odds of severe hunger (OR 3⋅01, 95 % CI 1⋅58, 5⋅74) among users who were separated or divorced/widowed was higher than the odds of moderate hunger (OR 2⋅71, 95 % CI 1⋅50, 4⋅90) while the odds of moderate hunger (OR 2⋅19, 95 % CI 1⋅23, 3⋅83) among single/never married pantry users was slightly larger than the odds of severe hunger (OR 2⋅01, 95 % CI 1⋅49–2⋅72) when compared with those who were married. Several variables were associated with only severe hunger, including working status (homemaker, disabled and retired) and monthly income ($1500–$1999, $500–$999 and less than $500). In addition, SNAP (0⋅53, 95 % CI 0⋅32, 0⋅88), WIC (0⋅20, 95 % CI 0⋅05, 0⋅78) and food pantry use in the past 30 d (0⋅44, 95 % CI 0⋅29, 0⋅69) were all found to be significantly protective against severe hunger. Pantry users who experienced any economic hardship had 1⋅95 times the odds of moderate hunger (95 % CI 1⋅10, 3⋅48). This association was much higher for severe hunger with OR 4⋅78 (95 % CI 2⋅49, 9⋅19).

**Table 3.** Adjusted mixed-effects models assessing associations between characteristics and ordinal hunger in food pantry users in ten pantries in Eastern Massachusetts, *n* 611

Variable	Moderate hunger vs. little to no hunger^a^^,^^b^		Severe hunger vs. little to no hunger^a^^,^^b^	
	Odds ratio (95 % CI)	*P*	Odds ratio (95 % CI)	*P*
Age^c^	0⋅98 (0⋅96, 1⋅01)	0⋅21	0⋅97 (0⋅94, 1⋅00)	**0**⋅**05**
Gender				
Male	Ref^d^		Ref^d^	
Female	1⋅34 (0⋅84, 2⋅13)	0⋅21	0⋅75 (0⋅53, 1⋅08)	0⋅12
Race/Ethnicity^e^				
Non-Hispanic White	Ref^d^		Ref^d^	
Non-Hispanic Black	1⋅76 (0⋅67, 4⋅62)	0⋅25	1⋅76 (0⋅56, 5⋅52)	0⋅33
Non-Hispanic Other	1⋅11 (0⋅55, 2⋅23)	0⋅77	0⋅99 (0⋅54, 1⋅80)	0⋅98
Hispanic	0⋅99 (0⋅50, 1⋅97)	0⋅97	0⋅94 (0⋅51, 1⋅74)	0⋅84
Educational Attainment				
College Graduate (4 years) or more	Ref^d^		Ref^d^	
High school or some college	1⋅58 (0⋅74–3⋅38)	0⋅24	1⋅63 (0⋅64–4⋅18)	0⋅30
Less than high school	2⋅37 (1⋅11–5⋅06)	**0**⋅**03**	1⋅97 (1⋅12–3⋅47)	**0**⋅**02**
Marital status				
Married/Living with partner	Ref^d^		Ref^d^	
Separated or Divorced/Widowed	2⋅71 (1⋅50–4⋅90)	**<0**⋅**01**	3⋅01 (1⋅58–5⋅74)	**<0**⋅**01**
Single/Never Married	2⋅19 (1⋅23–3⋅83)	**<0**⋅**01**	2⋅01 (1⋅49–2⋅72)	**<0**⋅**01**
Work status				
Full-time (>35 h)	Ref^d^		Ref^d^	
Part-time (<35 h)	0⋅92 (0⋅34–2⋅43)	0⋅86	1⋅53 (0⋅42–5⋅59)	0⋅52
Homemaker	1⋅04 (0⋅57–1⋅91)	0⋅89	2⋅25 (1⋅02–4⋅99)	**0**⋅**05**
Unemployed	0⋅91 (0⋅21–3⋅96)	0⋅90	1⋅18 (0⋅49–2⋅88)	0⋅71
Disabled	1⋅16 (0⋅45–3⋅02)	0⋅76	1⋅99 (1⋅09–3⋅63)	**0**⋅**02**
Retired	1⋅34 (0⋅64–2⋅80)	0⋅43	2⋅40 (1⋅18–4⋅87)	**0**⋅**02**
Other	0⋅36 (0⋅11–1⋅17)	0⋅09	0⋅92 (0⋅29–2⋅96)	0⋅89
Monthly household income^f^				
$2000 or more	Ref^d^		Ref^d^	
$1500 to $1999	1⋅39 (0⋅58–3⋅32)	0⋅45	2⋅39 (1⋅22–4⋅71)	**0**⋅**01**
$1000 to $1499	1⋅19 (0⋅51–2⋅82)	0⋅69	1⋅36 (0⋅53–3⋅49)	0⋅52
$500 to $999	1⋅12 (0⋅39, 3⋅26)	0⋅83	2⋅63 (1⋅82–3⋅81)	**<0**⋅**01**
Less than $500	2⋅09 (0⋅84–5⋅19)	0⋅11	3⋅70 (1⋅82–7⋅52)	**<0**⋅**01**
Household size^g^				
1	Ref^d^		Ref^d^	
2–3	1⋅61 (0⋅97–2⋅67)	0⋅07	0⋅7020 (0⋅43–1⋅14)	0⋅15
4–5	1⋅96 (0⋅96–3⋅99)	0⋅07	1⋅66 (0⋅61–4⋅54)	0⋅32
>5	0⋅56 (0⋅37–0⋅85)	**<0**⋅**01**	0⋅58 (0⋅36–0⋅93)	**0**⋅**02**
Household composition^h^				
Kids in the household	0⋅87 (0⋅54–1⋅39)	0⋅55	0⋅82 (0⋅51–1⋅31)	0⋅41
Seniors in the household	1⋅09 (0⋅48–2⋅45)	0⋅84	1⋅16 (0⋅57–2⋅37)	0⋅67
Food assistance use in the past 30 d^i^				
SNAP	0⋅82 (0⋅46–1⋅45)	0⋅50	0⋅53 (0⋅32–0⋅88)	**0**⋅**01**
WIC	0⋅75 (0⋅38–1⋅46)	0⋅39	0⋅20 (0⋅05–0⋅78)	**0**⋅**02**
Food pantry^j^	0⋅90 (0⋅58–1⋅40)	0⋅64	0⋅44 (0⋅29–0⋅69)	**<0**⋅**01**
Any economic hardship^k^	1⋅95 (1⋅10–3⋅48)	**0**⋅**02**	(2⋅49–9⋅19)	**<0**⋅**01**

Analyses were conducted using multivariate mixed-effects models for an ordinal outcome, with significance 0⋅05 indicated by boldface. The reference group for these analyses is little to no hunger in the household.

a Hunger categories were defined as little to no hunger in the household (HHS score 0–1), moderate hunger in the household (HHS score 2–3) and severe hunger in the household (HHS 4–6) according to the HHS score.

b The models controlled for all covariates in this model as fixed effects and food pantry sites was controlled for as a random effect.

c Age categories were created based on pre-established age definitions from the US Census.

d Socio-demographic categories that previous studies have found to be protective against food insecurity were used as the reference category.

e Individuals self-reported their race according to the pre-defined response options of Hispanic, non-Hispanic White, non-Hispanic Black and non-Hispanic Other.

f Income categories were created based on open-ended responses for annual/monthly income.

g Household size categories were created based on the open-ended responses of the number of people in household.

h Household composition for both children and seniors in the household were defined as at least one or more in the household.

i Food assistance use (SNAP, WIC, food pantry) were defined as any use in the past 30 d, with no use as the comparison.

j Food pantry use was defined as use of a food pantry in the past 30 d aside from the visit of survey completion.

k Economic hardship defined as experiencing at least one of the following in the past 3 months: medical expenses, job loss, reduced pay/hours, divorce, rent or utilises, foreclosure/eviction notice, death of a family member or breadwinner, business financial difficulty, home repairs, loan repayment or loan late fees, legal expenses or other hardship.

## Discussion

More than a third (39⋅27 %) of food pantry users reported experiencing moderate or severe hunger despite the majority recently using charitable and federal food assistance; 87⋅73 % reported using food pantries in the past month and 54⋅83 % reported using SNAP in the past month. Factors associated with hunger included being divorced, separated or widowed and being single/never married; less than a high school level of education; SNAP, WIC and food pantry use; and experience of any economic hardship in the previous 3 months. Marital status, specifically being single or separated, and divorced or widowed were associated with both moderate and severe hunger after adjusting for other factors. Educational attainment of less than a high school level of education was significantly associated with severe hunger. Economic hardship had the strongest association with severe and moderate hunger, which is especially important given the current context. While this study was conducted prior to the COVID-19 pandemic, economic hardship has been pronounced since the pandemic and will continue to be experienced by many^(5)^.

There are a number of federal food assistance programmes in the US. Some programmes are specifically targeted to high-risk populations, such as seniors and children, while others more broadly include all low-income eligible populations^(32)^. Each has a different way of administering food with some providing financial assistance to purchase groceries and others providing on-site meals. These programmes and policies have been in existence pre-pandemic but some were adapted to respond to conditions exacerbated by the pandemic specifically through the American Rescue Plan, which provides $12⋅5 billion to reduce food insecurity caused by the pandemic^(33)^. For example, during the pandemic the National School Breakfast Program and National School Lunch Program, which previously required determination of free- or reduced-price eligibility based on income status, were made freely available to all school-aged children.

Given the availability of local and national resources, even before the pandemic, individuals experiencing hunger and food insecurity were accessing these programmes. For example, SNAP usage across the US was 12 % in 2019 but in this sample of food pantry users, it was almost four times as much and yet 20⋅13 % of food pantry users reported moderate hunger and 19⋅14 % reported severe hunger^(34)^. The majority of food pantry users in this study earned less than $1500 per month (72⋅18 %) and experienced at least one economic hardship (60⋅39 %) and still reported high levels of hunger even as they accessed resources to reduce it. The need for more government and charitable financial and food assistance may increase in the aftermath of the pandemic with decreased incomes and increased economic hardships and the barriers to accessing these programmes will need to be addressed^(21)^.

Low income and the inability to afford food is the major cause of food insecurity^(8)^. The majority of food pantry users in this study earned less than $1500 per month (72⋅18 %) and experienced at least one economic hardship (60⋅39 %). Consistent with other research, we found an association between income and hunger. In addition, prior studies found that major changes in financial strain are also associated with food insecurity^(12)^. In our study, the odds of severe hunger (OR 4⋅78, 95 % CI 2⋅49, 9⋅19) was higher than the odds of moderate hunger for participants that experienced any economic hardship in the past 3 months compared with those that did not. Income and economic hardship have been established as highly correlated with food insecurity; hunger is greatly impacted by the need to make financial tradeoffs and lack of funds results in financial strain, which can often lead to reduced correlating to spending on food^(13,35,36)^.

The odds of severe hunger were greater for homemakers, retirees and disabled pantry users compared with the odds for unemployed individuals, which is inconsistent with prior research that found unemployed individuals have a higher prevalence of food insecurity^(5)^. While unemployment may play a role in food security status, our findings suggest that other factors, not strictly unemployment, may be indicative of hunger status. This will be important to consider as the pandemic has resulted in changes to employment status through permanent and temporary layoffs^(35,37)^.

Previous research found having children in the household is significantly associated with increased hunger^(38,39)^ and in 2020, the percent of children experiencing very low food security and disrupted eating increased from 0⋅6 % to 0⋅8 %^(5)^. Our study, however, did not find a significant relationship in adjusted models between children in the household and a higher risk of hunger. This could be due in part due to children's access to food assistance programmes, such as free and reduced-price school meals, SNAP or WIC, which have been shown to alleviate food insecurity^(40–44)^. In our study, the use of SNAP, WIC and food pantries were all found to be protective against severe hunger.

A major strength of our study was the inclusion of the modified, validated HHS and the ability to quantify levels of hunger. The HHS is short and was easily embedded in our survey to allow for the quantification of hunger, which is often difficult to measure as an individual psychological condition^(1)^. The tool allows for a multicultural comparison of food deprivation in a variety of contexts^(27–30)^. Although most hunger assessments are administered in low-income countries, the HHS was adapted to be used in a range of populations^(27–30)^. Assessing hunger in high-income countries is not as common as assessing food insecurity; however, measures are still needed when conducting research or surveillance in highly food insecure populations. The present study suggests a method of measuring hunger for monitoring and surveillance and to understand the determinants of hunger to determine populations that may have additional assistance needs^(28)^.

Our study also had several limitations. First, the sample size and socio-demographic composition of food pantry users varied greatly across study sites, though our mixed-effects models accounted for these differences. There are factors at the level of the food pantry (i.e. amount of food distributed, number of households served, length of time food pantry has operated) that were not controlled for in analyses that may limit the external validity of the findings. We used mixed-effects modelling to control for food pantry, which did control for some factors that differed across food pantries (i.e. socio-demographic factors of food pantry clients). Second, the study population consisted of a self-selected sample of pantry users who volunteered to participate and met the inclusion criteria of speaking English or Spanish, limiting the study's generalisability to pantries that serve similar populations. We found, however, that food pantry users in our study represented a range of characteristics and experiences that make our findings informative for a wide variety of populations in suburban and urban areas of the US. Third, there may have been recall bias as individuals who were more likely to report potentially negative determinants of hunger, such as economic hardships, may also be more likely to report experiencing hunger. Fourth, hunger and food insecurity can be negatively stigmatised and may lead to social desirability bias with individuals underreporting their experiences of hunger. Finally, our study design does not allow for conclusions on causal relationships between determinants and hunger given it is a cross-sectional study.

The present research provides insight into the determinants of hunger among food pantry users and offers a practical way to measure hunger in food pantry users. While several determinants were found to be associated with household hunger status in food pantry users, experiences of recent economic hardship were found to have the strongest relationship with hunger. This is important as the economic context for many has changed due to the COVID-19 pandemic^(33)^ and will continue to shift in the aftermath including job loss, unexpected medical or other expenses, and loss of stable housing.

Understanding determinants of hunger pre-pandemic, and how those determinants have been impacted by the pandemic, could better inform future emergency responses to address hunger. For example, a detailed exploration on the types of economic hardship experienced could provide insight to more targeted financial assistance programmes and hunger relief efforts. Examination of food assistance programme usage in food pantry users can inform enhancement of existing efforts as well as the development of new ones to address gaps in services and underutilisation by those who qualify^(34)^. In particular, efforts should focus on enrolment and retention in food assistance programmes for populations that experience moderate or severe hunger including those with low educational attainment, pre-existing use of food assistance programmes, and other socio-demographic conditions through targeted promotion and communication approaches. In addition, a focus on strengthening already existing food assistance programmes including food pantries, SNAP and WIC, through additional funding, streamlined enrolment processes, targeted promotional efforts and de-stigmatizing participation are warranted.

Ultimately, being able to easily and quickly measure and monitor hunger of those in need will be crucial to developing interventions, implementing solutions, monitoring success and informing adaptations to sustain them over time with changing environmental influences. Future research in this area should continue to explore the determinants affecting hunger, using a brief and easily implementable tool to measure it, as well as evaluate the efforts to address hunger in new and existing users of government and charitable food assistance programmes.
